# HIV Protease Inhibitors and Insulin Sensitivity: A Systematic Review and Meta-Analysis of Randomized Controlled Trials

**DOI:** 10.3389/fphar.2021.635089

**Published:** 2021-11-01

**Authors:** Violet Dismas Kajogoo, Mary Gorret Atim, Demeke Amare, Melka Geleta, Yilkal Muchie, Hanna Amanuel Tesfahunei, Willyhelmina Olomi, Joan Acam, Tsegahun Manyazewal

**Affiliations:** ^1^ Center for Innovative Drug Development and Therapeutic Trial for Africa (CDT-Africa), College of Health Sciences, Addis Ababa University, Addis Ababa, Ethiopia; ^2^ Mafia District Hospital, Mafia Island, Tanzania; ^3^ Busitema University Faculty of Health Sciences, Mbale, Uganda; ^4^ Ethiopian Food and Drug Administration Authority (EFDA), Addis Ababa, Ethiopia; ^5^ Federal Ministry of Health, Addis Ababa, Ethiopia; ^6^ All Africa TB Leprosy Training and Rehabilitation (ALERT) Center, Addis Ababa, Ethiopia; ^7^ Hager Biomedical Research Institute, Asmara, Eritrea; ^8^ NIMR–Mbeya Medical Research Program (MMRP), Mbeya, Tanzania; ^9^ Pope Johns Hospital – ABER, Lira Municipality, Uganda

**Keywords:** HIV, protease inhibitors, insulin resistance, diabetes, antiretrovirals

## Abstract

**Background:** Protease inhibitors (PIs) are believed to affect insulin sensitivity. We aimed to analyze the effect of PIs on insulin sensitivity and the onset of diabetes mellitus (DM) in patients with HIV.

**Methodology:** We searched PubMed, Google Scholar, ClinicalTrals.gov, and the WHO International Clinical Trials Registry Platform till November 2020 for randomized controlled trials (RCTs) that studied the effects of PIs on insulin sensitivity and DM in patients with HIV. We followed the PRISMA and PICOS frameworks to develop the search strategy. We used the random-effects meta-analysis model to estimate the mean difference (MD), standardized mean difference (SMD), and risk ratios for our outcomes, using Stata 14 software.

**Results:** We included nine RCTs that enrolled 1,000 participants, with their ages ranging from 18 to 69 years. The parameters and investigations used in the studies to determine insulin sensitivity were glucose disposal rates, hyperglycemia, and mean glucose uptake. The majority of results showed an association between PIs and insulin sensitivity. The pooled analysis showed no statistically significant difference in insulin sensitivity with atazanavir, whether the study was performed on healthy individuals for a short term or long term in combination with other drugs like tenofovir or emtricitabine [SMD = 0.375, 95% CI (0.035, 0.714)]. The analysis showed reduced glucose disposal rates and hence reduced insulin sensitivity with lopinavir (heterogeneity chi-squared = 0.68, I-squared [variation in SMD attributable to heterogeneity] = 0.0%, *p* = 0.031). The heterogeneity with chi-squared was substantial (61–80%), while with I-squared was not significant (0–40%), *p* = 0.031). Less adverse events were observed with atazanavir than with lopinavir [RR = 0.987, 95% CI (0.849, 1.124)]. Darunavir and indinavir did not demonstrate any significant changes in insulin sensitivity. Most of the studies were found to have a low risk of bias.

**Conclusions:** There are significant variations in the effects of PIs on insulin sensitivity and onsets of DM. Atazanavir, fosamprenavir, and darunavir did not demonstrate any significant changes in insulin sensitivity, compared to the rest of the group. There is a need to assess the benefits of PIs against the long-term risk of impaired insulin sensitivity. All patients newly diagnosed with HIV should have DM investigations before the start of ARVs and routinely. RCTs should focus on sub-Saharan Africa as the region is worst affected by HIV, but limited studies have been documented.

## Introduction

The human immunodeficiency virus (HIV) is a retrovirus that carries a single-stranded RNA as its genetic material, with high mutation rates that often lead to viral escape from multiple drugs (Fidler et al., 2020; Saag et al., 2020). A variety of antiretrovirals (ARVs) have been developed that highly suppress HIV replication, while there has been no effective cure. According to the World Health Organization 2021 global progress report on HIV (World Health Organization, 2021), an estimated 3.7 million people were living with HIV at the end of 2020, of whom two-thirds were in Africa. In the same year, 68,000 people died due to HIV and 1.5 million people acquired new HIV infection (World Health Organization, 2021). Since 2016, the WHO has recommended a lifelong antiretroviral treatment for all people living with HIV, including children and pregnant women, regardless of the CD4 count or clinical status (World Health Organization (WHO), 2016). This eventually improved the survival of patients (Chang et al., 2020) but lifted chronic complications in clinical practice (Bygrave et al., 2020).

Metabolic disorders such as diabetes mellitus (DM) have been associated with HIV and ARVs (Echecopar-Sabogal et al., 2018; Sarfo et al., 2021), and carbohydrate metabolism abnormalities like insulin resistance, hyperglycemia, and DM have been associated with ARVs of the class protease inhibitors (PIs) (Noor et al., 2006). Some earlier PIs such as indinavir and lopinavir/ritonavir exasperate lipid profiles and increase the risk of developing insulin resistance, hyperglycemia, and DM (Overton et al., 2016). PIs also mediate the blockage of glucose transport. In non-HIV metabolic syndrome, insulin resistance is further integrated with endoplasmic reticulum and oxidative stresses, lipotoxicity and lipid metabolism disruption, and altered adipocytokine secretion (Hruz, 2011; Zha et al., 2013).

In preclinical studies, PIs altered glucose transporter 4 (GLUT4)—one of the most important glucose transporters—decreased insulin secretion by affecting β-cells, thereby increasing insulin resistance (Hertel et al., 2004; Vyas et al., 2010; Monroe et al., 2015). The clinical outcomes and the overall impacts of ARV initiations on glucose metabolism remain ambiguous (Erlandson et al., 2014; Monroe et al., 2015). Several studies have evaluated the development of DM when PIs are administered in combination or with other classes of ARVs. In this particular review, we aimed to compare PIs towards their effects on insulin sensitivity and the onset of diabetes mellitus (DM) in patients with HIV.

## Methods

The review followed the Preferred Reporting Items for Systematic Reviews and Meta-Analyses (PRISMA) guidelines (Shamseer et al., 2015). We compared PIs separately and looked at their effects on insulin sensitivity and determined if the findings can be used for the present and future in the creation of a proper combination of ARVs for patients with HIV and comorbidity of DM.

### Search Methods

We searched PubMed, Google Scholar, ClinicalTrals.gov, and the WHO International Clinical Trials Registry Platform for completed randomized controlled trials (RCTs) up to November 1, 2020. Medical Subject Headings (MeSH) (Baumann, 2016) was used to develop search terms, using combined key terms that we drove from our research questions. For PubMed, Boolean operations were used in between search terms like protease inhibitors and hyperglycemia or protease inhibitors and insulin sensitivity or protease inhibitors and cholesterol. References of the retrieved articles were also searched to identify other similar articles.

### Search Strategy

PubMed MeSH terms used were “insulin sensitivity” [Text Word] OR “insulin”[MeSH Terms] OR (“insulin resistance, Type 2”[Mesh]) AND “Protease inhibitors”[MeSH Terms] OR “HIV protease inhibitors” [Text Word] OR “Lopinavir” [Text Word] OR “Atazanavir” [Text Word] OR “Darunavir” [Text Word] AND “Antiretroviral” [Text Word] OR “Antiretroviral Agents”[Mesh] OR “Antiretroviral Therapy,” OR “Highly Active”[Mesh] AND “Incidence”[Text Word] OR “Incidence”[Mesh] AND “Epidemiology”[Mesh] OR “epidemiology” [Subheading] AND “Incidence”[Mesh].

### Eligibility Criteria

PICOS (participants, interventions, comparison, outcomes, and study design) (Gebrie et al., 2020) was used to formulate the eligibility criteria.

- Population: individuals with HIV.
- Intervention: PIs.
- Comparison: PIs of different classes.
- Outcome:
- Primary outcome: effects on insulin sensitivity.
- Secondary outcomes: adverse events.
- Study design: RCTs published from 2000 to November 1, 2020.

### Inclusion Criteria

We used the Jadad scale with three main characteristics of description (RCT, blinding, withdrawal, and dropouts). We considered RCTs that studied any drug in the PI group and conducted in any part of the world. We considered studies with HIV-seronegative participants (phase 1) and with seropositive participants but not diagnosed with DM that evaluated one or more of the outcomes listed before and of English language.

### Exclusion Criteria

We excluded studies that had patients with DM at baseline, those that had combined therapies of other ARTs, and those not published in peer-reviewed publisher/journals or not yet completed.

### Study Selection

Published studies were needed to include information on the interaction between PIs and insulin sensitivity (Béïque et al., 2007) based on the pre-identified criteria. We imported articles from the electronic databases into STATA software 14, screened for titles, followed by the abstracts and full text by two reviewers. Any discrepancies in findings between the two reviewers were discussed and resolved. All studies were RCTs written in English. Before the screening, we identified and removed redundant papers.

### Data Extraction, Synthesis, and Analysis

Data were extracted by two independent reviewers using a data extraction tool adopted from the Cochrane Library (Noyes et al., 2018). This included the author’s name, article publication year, study country, study design, number of participants, length of follow-up, intervention PIs, outcomes, and adverse events. Data from eligible studies were transferred to a spreadsheet on Microsoft Excel. Studies that had designs and interventions that were alike, with assessments of the same outcomes and sufficient enough data, were used to perform the meta-analysis. Articles were summarized using a clinical-based approach (Siwek et al., 2002).

### Assessment of Risk of Bias

The risk of bias was assessed using the revised Cochrane risk of bias tool for randomized trials (RoB 2) (Sterne et al., 2019). Two authors independently judged these risks as low, unclear, or high based on critical domains including random sequence generation, allocation concealment, blinding, incomplete outcome data, and selective reporting.

### Statistical Analysis

STATA was used for statistical analysis. The random-effects model was used in performing the meta-analysis to estimate the mean differences and standardized mean difference for continuous outcomes and risk ratio for dichotomous outcomes of adverse events, with 95% CI. Data were analyzed according to the intention-to-treat principle (Sedgwick, 2015), and heterogeneity was measured using the chi-squared and I^2^ statistics.

### Operational Definitions

ARV: antiretroviral drugs used to treat HIV.
ART: a combination of antiretroviral drugs for the treatment of HIV.
Seronegative patients: patients who have tested negative for HIV.
Patients with highly active ART failure: patients on ARTs having at least 1,000 and more copies of HIV viral load.
Phase 1 trial: the earliest trial carried out to test HIV treatment for the first time in a small group of people.
Biomarker: the characteristics that is measured as an indicator of responses to an HIV treatment.

## Results

### Search Results

**FIGURE 1:**
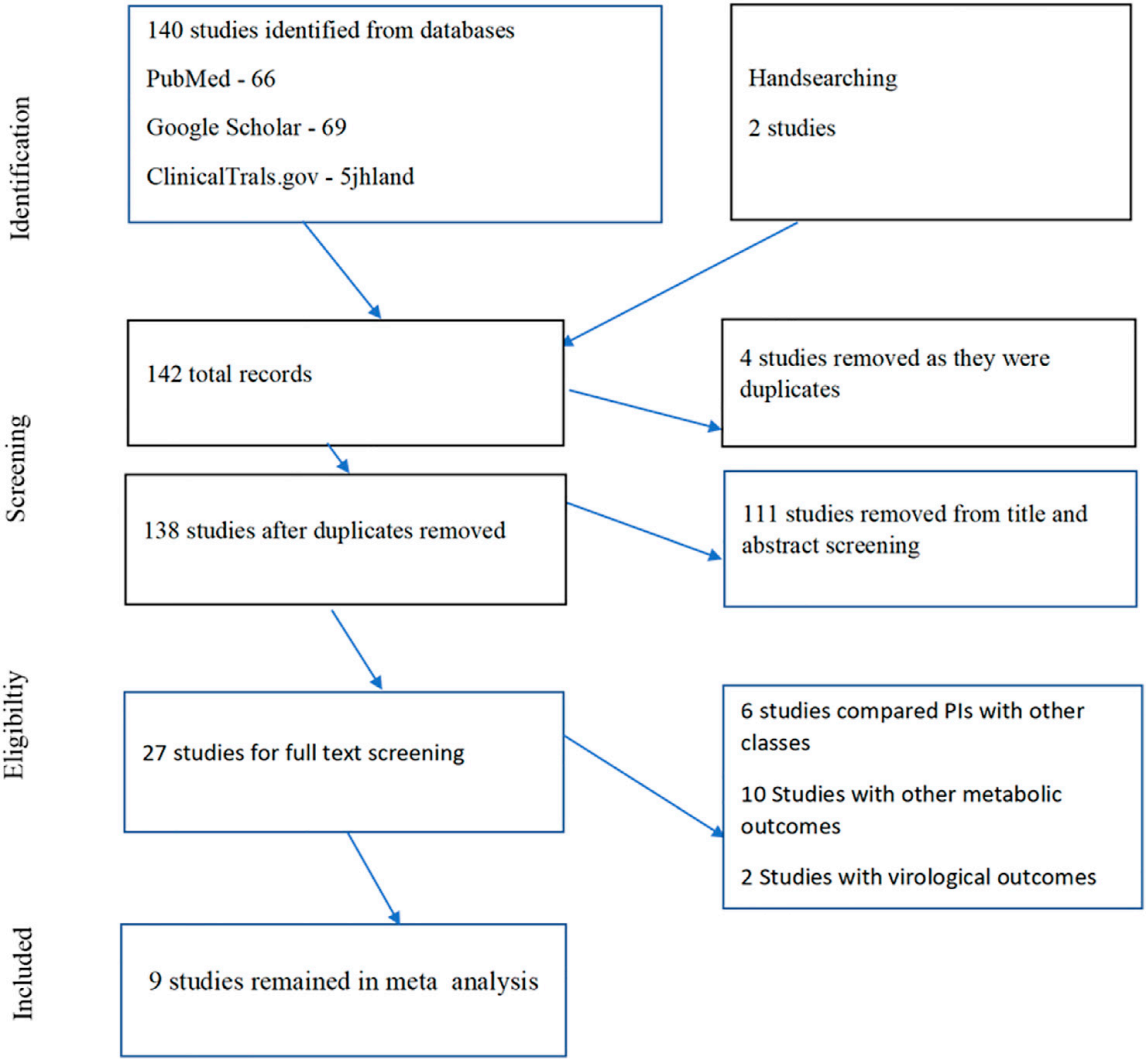
Study flow diagram.

We identified 142 studies, with four articles removed as they were duplicates and 111 studies removed because they did not meet the inclusion criteria (Figure 1). We reviewed 27 full-text studies and finally left with nine studies. The selected studies were mostly from the United States, and one multinational study included Europe and Latin America, with people of different ethnicities (Black, White, Latinas, and Caucasians).

### Study Characteristics

Table 1 presents a summary of the included nine RCTs. The studies included 1,000 adults with ages ranging from 18 to 69 years.

**TABLE 1:** Characteristics of included RCTs.

Author, year	Country	Study design	Participants number	Follow-up duration	Intervention drugs	Treatment outcomes	Adverse events
Overton et al. (2016)	The United States	Phase 4 randomized trial	22	48 weeks	DRV/r	9.9	2/12
					ATV/r	9.1	1/10
						Glucose disposal rate	
Noor et al. (2004)	The United States	Randomized, double-blind, crossover study	30	5 days	Placebo	9.88	None
					ATV	9.80	
					LPV/r	7.52	
						Glucose disposal rate	
Noor et al. (2006)	The United States	Randomized, crossover study	26	10 days	ATV/r	10.4	None
					LPV/r	8.6	
						Glucose disposal rate	
Lee et al. (2007)	The United States	Randomized, double-blind, crossover study	14	28 days	RTV	8.0	1/8
					Amprenavir	8.4 glucose disposal rate	0/6
Molina et al. (2010)	Multicenter	Multicenter, open-label, non-inferiority randomized trial	883	96 weeks	ATV/RTV	3/434	283
					LPV/RTV	2/428	282
						Hyperglycemia	
Stanley et al. (2009)	The United States	Randomized non-blinded trial	12	6 months	ATV/RTV	26.7	3/5
					LPV/RTV	24.4	1/7
						Glucose disposal rate	
Dubé et al. (2008)	The United States	Placebo-controlled trial	30	4 weeks	ATV/RTV	6.73	
					LPV/RTV	8.88	
					Placebo	7.53	
						Mean glucose uptake	
Noor et al. (2002)	The United States	Randomized placebo-controlled trial	06	7–10 days	IDV	13.5	
					Placebo	14.1	
						Glucose disposal rate	
Randell et al. (2010)	The United Kingdom	Randomized trial	27	2 weeks	FPV	4.48	
					LPV	0.28	
						M/I (ratio of glucose disposal rate)	

DRV, darunavir; ATV, atazanavir; IDV, indinavir; RTV, ritsonavir; LPV, lopinavir; FPV, fosamprenavir.

### The Risk of Bias and Methodological Quality

**FIGURE 2:**
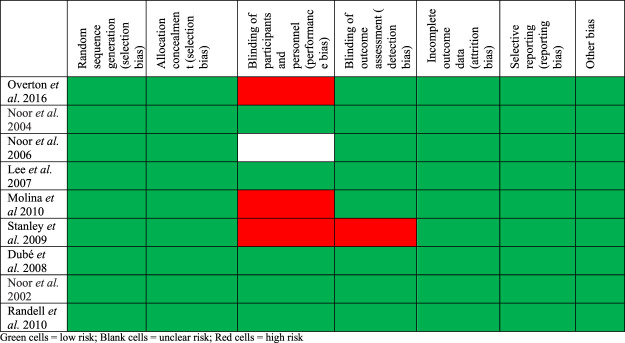
Risk of bias summary: review authors’ judgments about each’ risk of bias’ item for included trials.

Figure 2 summarizes what the authors reviewed and their judgments about each risk of bias for included trials. Most of the studies were found to have a low risk of bias. Four of the studies did not have any bias at all. The studies by Overton et al. (2016) and Molina et al. (2010) were found with bias due to the blinding of participants as they were single-blinded trials. The study by Stanley et al. (2009) was an open-labeled trial, hence increasing its level of bias.

### Glucose Disposal Rates Post-Intervention

Table 2 summarizes the glucose disposal rates post-intervention. A euglycemic clamp was used for all the aforementioned studies. The gold standard for assessing insulin resistance in humans is the hyperinsulinemic–euglycemic clamp. Overton et al. (2016) and Stanley et al. (2009) were open-labeled studies, while the rest were double-blinded studies.

**TABLE 2:** Glucose disposal rate post-intervention.

Trial	Atazanavir	Lopinavir	Darunavir	Ritonavir	Amprenavir	Placebo	Indinavir
Overton et al. (2016)	9.1	—	9.9				
Noor et al. (2004)	9.80	7.52					
Noor et al. (2006)	10.4	8.6					
Lee et al. (2007)	—	—	—	8.0	8.4		
Stanley et al. (2009)	26.7	24.4					
Dubé et al. (2008)	6.73	8.88	—	—	—	7.53	
Noor et al. (2002)	—	—	—	—	—	14.1	13.5

Seven studies reported glucose disposal rates with different PIs (Table 2). From the observations, we notice the IDV placebo study has the highest glucose disposal. There were no statistically significant differences in baseline fasting body weight, plasma glucose, insulin, lipid, and lipoprotein levels between placebo- and indinavir-treated subjects (Noor et al., 2002). The euglycemic–hyperinsulinemic clamp duration was 3-h, which did not show a difference from other studies (most were between 3 and 3.5 h). DRV as compared to ATV had a higher glucose disposal rate. Lee et al. (2007) compared RTV and amprenavir, which had a slight statistical difference in glucose disposal rates. One study, Randell et al. (2010), calculated the insulin sensitivities between FPV and LPV by M/I (glucose disposal rates over the mean insulin concentrations). There was decreased insulin sensitivity with patients on LPV.

**FIGURE 3:**
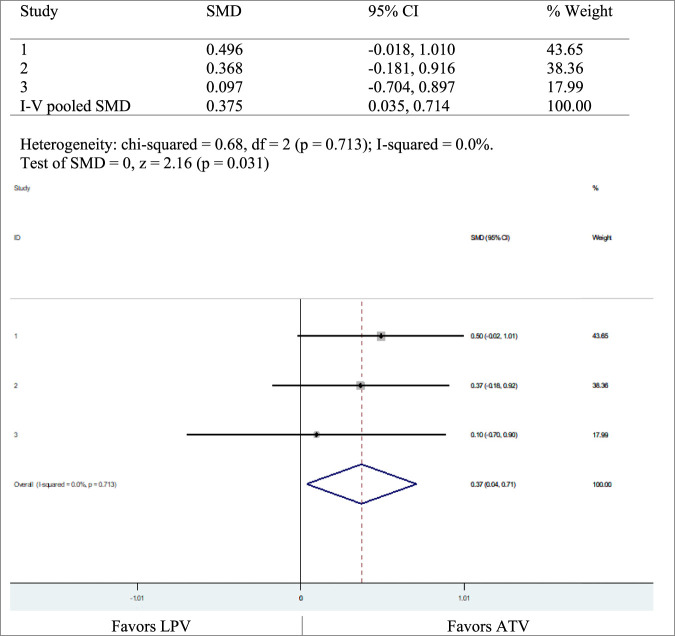
Glucose disposal rate, LPV versus ATV.

Three studies, Noor et al. (2004), Noor et al. (2006), and Stanley et al. (2009), compared ATV and LPV with variable results. We carried out a meta-analysis, showing that the glucose disposal rates were in favor of ATV (Figure 3).

### Adverse Events

**FIGURE 4:**
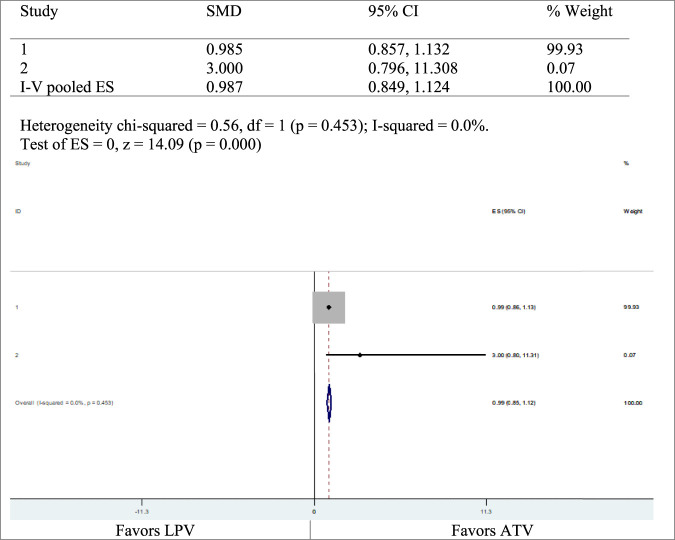
Adverse events of ATV and LPV.

Table 3 summarizes the adverse events observed. Two studies reported AEs of ATV and LPV. The first study had a risk ratio of 0.9 with 95% CI (0.8574586–1.131936), and the second study had a risk ratio of 3 with 95% CI (0.7958937–11.30804) (Figure 4).

**TABLE 3:** Adverse events.

Author, year	Country	Risk ratio	Lower confidence interval	Upper confidence interval
Molina et al. (2010)	Multicenter	0.9851843	0.8574586	1.131936
Stanley et al. (2009)	The United States	3	0.7958937	11.30804

## Discussion

Nine of the included RCTs studied the effects of PIs on insulin sensitivity. Insulin sensitivity did not show significant changes with ATV, whether the study is performed on healthy individuals for short term like 5, 10, or 28 days (Noor et al., 2004; Overton et al., 2016) or on HIV-infected individuals for a long term in combination with other drugs like tenofovir and emtricitabine parallelly. LPV reduced glucose disposal rates and hence reduced insulin sensitivity. This was supported by a meta‐analysis of the three studies [heterogeneity chi-squared = 0.68, I-squared (variation in SMD attributable to heterogeneity) = 0.0%, *p* = 0.031]. The heterogeneity with chi-squared was substantial (61–80%), while that with I-squared was not significant (0–40%), *p* = 0.031). Darunavir does not demonstrate any significant changes in insulin resistance in HIV-infected individuals, on long-term use in combination with other drugs (Overton et al., 2016). The plasma glucose levels are within normal ranges when using amprenavir as demonstrated by healthy individuals for a short term of 28 days (Lee et al., 2007). RTV and LPV increase plasma glucose levels in healthy individuals on acute use and increase insulin sensitivity (Noor et al., 2004). A meta-analysis on two studies, Molina et al. (2010) and Stanley et al. (2009), showed that there was a higher risk of developing adverse events on the LPV arm than on the ATV arm, with heterogeneity chi-squared = 0.56, *p* = 0.453 and I-squared (variation in ES attributable to heterogeneity) = 0.0%, *p* = 0.000; chi-squared heterogeneity was moderate (41–60%) while I-squared was not significant (0–40%). For treatment of adults with HIV, the current WHO guidelines for ART recommend two nucleoside reverse transcriptase inhibitors (NRTIs) and one non-nucleoside reverse transcriptase (NNRTI) or an integrase inhibitor (INSTI) consisting of the first-line drugs (World Health Organization (WHO), 2016). In special situations and circumstances, abacavir (ABC) or boosted PIs (ATV/r, DRV/r, LPV/r) are an option (World Health Organization (WHO), 2016).

Evaluating the quality of included studies, we noticed that standard biomarkers were used in all the trials. RCTs were mainly used and emphasized as they are most likely to be representative of the true difference in the comparison arms. In rare events, it is statistically limited to detect the power by use of RCTs. Some studies were conducted on persons with no ART experience, while other studies considered ART-experienced patients, with a history of treatment failure and HIV-seronegative; these could have led to recruitment bias. The sample sizes of most studies were small, with less than 300 participants, and short study duration (48 weeks and less), which could have led to selection bias.

Randell et al. (2010) demonstrated effects using both euglycemic clamp and HOMA IR. The results of both analyses correlate with each other, showing no significant changes. In a cross-sectional study in Angola (Francisco et al., 2020), insulin resistance based on the homeostatic model (HOMA-R) was 20% and glucose intolerance was 40%. Two other cross-sectional analyses of longitudinal cohort and cohort studies conducted in Italy (Rosso et al., 2007) and Rwanda (Dusingize et al., 2013) compared median HOMA IR and fasting glucose. The first study (Rosso et al., 2007) demonstrates that FPG was in normal ranges (78.5), while HOMA IR was significantly high (2.18). The second study (Dusingize et al., 2013) reports that the median for both is in normal ranges (FPG = 77.5, HOMA IR = 0.66). A cross-sectional study conducted in the United States (Tiozzo et al., 2021) compared results of HOMA IR and HbA1c for patients with a shorter duration of fewer than 15 years or a longer duration of greater than 16 years. For the shorter duration, the HOMA IR was above normal ranges, while HbA1c was normal (2.8 and 5.8, respectively). For a longer duration, the HOMA IR was above normal ranges, while HbA1c was normal (4.0 and 6.1, respectively). From the aforementioned analysis, we can conclude that more RCTs need to be done with a comparison of HOMA IR with the other methods (OsGTT, HbA1c). Treating patients with DM is complex due to the progressive nature of the disease (Gebrie et al., 2021a; Gebrie et al., 2021b), and the burden could be higher in HIV comorbidity (Godman et al., 2020; Ye et al., 2020; Lopez-Alvarenga et al., 2021; Phoswa, 2021) that calls for more robust studies in the area.

## Conclusion

In this review, there were significant variations in the effects of PIs on insulin sensitivity and onsets of DM. Atazanavir, fosamprenavir, and darunavir do not demonstrate any significant changes in insulin sensitivity, compared to the rest of the group. There is a need to assess the benefits of the PIs against the long-term risk of impaired insulin sensitivity. We recommend that all patients newly diagnosed with HIV should be investigated for DM and hyperglycemic factors, with the tests available in a particular setting before the start of ARTs and routinely. Close monitoring of the patients should be done in every visit for any signs and symptoms. From the aforementioned review, it is evident that just one class of drugs has different variations on the onset of DM in PLHIV. More studies should be conducted on the evaluation of not only PIs but also all ARV classes and the effects or onsets of DM. RCTs should focus on sub-Saharan Africa as the region is worse affected by HIV, but limited studies have been documented.

## Data Availability

The original contributions presented in the study are included in the article/Supplementary Material; further inquiries can be directed to the corresponding author.

## Abbreviations

AE, adverse events; AMP, amprenavir; ART, antiretroviral therapy; ARV, antiretrovirals; ATV, atazanavir; DM, diabetes mellitus; FBG/FPG, fasting blood glucose/plasma glucose; FPV, fosamprenavir; HAART, highly active antiretroviral therapy; HbA1c, glycated hemoglobin; HIV, human immunodeficiency virus; HOMA IR, homeostatic model; IDV, indinavir; INSTIs, integrase strand transfer inhibitors; LPV, lopinavir; MS, metabolic syndrome; NCD, non-communicable diseases; NRTI, nucleoside reverse transcriptase inhibitor; NNRTI, non-nucleoside reverse transcriptase; OGTT, oral glucose tolerance test; PI, protease inhibitors; PLHIV, people living with HIV; RBG, resting blood glucose; and RTV, ritonavir.
